# Seasonal variations in the diagnosis of childhood cancer in the United States

**DOI:** 10.1038/sj.bjc.6690729

**Published:** 1999-10

**Authors:** J A Ross, R K Severson, A R Swensen, B H Pollock, J G Gurney, L L Robison

**Affiliations:** Department of Pediatrics, University of Minnesota Division of Pediatric Epidemiology and Clinical Research, Minneapolis, MN, 55455, USA; University of Minnesota Cancer Center, Minneapolis, MN, USA; Karmanos Cancer Institute and the Department of Family Medicine, Wayne State University, 110 E Warren Avenue, Detroit, MI, 48201, USA; Pediatric Oncology Group, Statistical Office, Suite 22, Gainesville, FL, 32608-2516, USA

**Keywords:** seasonality, childhood cancer, leukaemia, infection

## Abstract

Seasonal trends in month of diagnosis have been reported for childhood acute lymphoblastic leukaemia (ALL) and non-Hodgkin's lymphoma (NHL). This seasonal variation has been suggested to represent an underlying viral aetiology for these malignancies. Some studies have shown the highest frequency of diagnoses in the summer months, although this has been inconsistent. Data from the Children's Cancer Group and the Pediatric Oncology Group were analysed for seasonal incidence patterns. A total of 20 949 incident cancer cases diagnosed in the USA from 1 January 1989 through 31 December 1991 were available for analyses. Diagnosis-specific malignancies available for evaluation included ALL, acute myeloid leukaemia (AML), Hodgkin's disease, NHL, rhabdomyosarcoma, neuroblastoma, retinoblastoma, osteosarcoma, Wilms' tumour, retinoblastoma, Ewings' sarcoma, central nervous system (CNS) tumours and hepatoblastoma. Overall, there was no statistically significant seasonal variation in the month of diagnosis for all childhood cancers combined. For diagnosis-specific malignancies, there was a statistically significant seasonal variation for ALL (*P* = 0.01; peak in summer), rhabdomyosarcoma (*P* = 0.03; spring/summer) and hepatoblastoma (*P* = 0.01; summer); there was no seasonal variation in the diagnosis of NHL. When cases were restricted to latitudes greater than 40° (‘north’), seasonal patterns were apparent only for ALL and hepatoblastoma. Notably, 33% of hepatoblastoma cases were diagnosed in the summer months. In contrast, for latitudes less than 40° (‘south’), only CNS tumours demonstrated a seasonal pattern (*P* = 0.002; winter). Although these data provide modest support for a summer peak in the diagnosis of childhood ALL, any underlying biological mechanisms that account for these seasonal patterns are likely complex and in need of more definitive studies. © 1999 Cancer Research Campaign


*British Journal of Cancer* (1999) **81**(3), 549–553
© 1999 Cancer Research Campaign
Article no. bjoc.1999.0729

### Seasonal variations in the diagnosis of childhood cancer in the United States

**JA Ross[1,2], RK Severson[3], AR Swensen[1], BH Pollock[4], JG Gurney[1,2] and LL Robison[1,2]**

[1]University of Minnesota Division of Pediatric Epidemiology and Clinical Research, Department of Pediatrics, Minneapolis, MN 55455, USA; [2]University of Minnesota Cancer Center, Minneapolis, MN, USA; [3]Karmanos Cancer Institute and the Department of Family Medicine, Wayne State University, 110 E Warren Avenue, Detroit, MI 48201, USA; [4]Pediatric Oncology Group, Statistical Office, Suite 22, 4110 SW 34th Street, Gainesville, FL 32608-2516, USA

**Summary** Seasonal trends in month of diagnosis have been reported for childhood acute lymphoblastic leukaemia (ALL) and non-Hodgkin's lymphoma (NHL). This seasonal variation has been suggested to represent an underlying viral aetiology for these malignancies. Some studies have shown the highest frequency of diagnoses in the summer months, although this has been inconsistent. Data from the Children's Cancer Group and the Pediatric Oncology Group were analysed for seasonal incidence patterns. A total of 20 949 incident cancer cases diagnosed in the USA from 1 January 1989 through 31 December 1991 were available for analyses. Diagnosis-specific malignancies available for evaluation included ALL, acute myeloid leukaemia (AML), Hodgkin's disease, NHL, rhabdomyosarcoma, neuroblastoma, retinoblastoma, osteosarcoma, Wilms' tumour, retinoblastoma, Ewings' sarcoma, central nervous system (CNS) tumours and hepatoblastoma. Overall, there was no statistically significant seasonal variation in the month of diagnosis for all childhood cancers combined. For diagnosis-specific malignancies, there was a statistically significant seasonal variation for ALL ($P$ = 0.01; peak in summer), rhabdomyosarcoma ($P$ = 0.03; spring/summer) and hepatoblastoma ($P$ = 0.01; summer); there was no seasonal variation in the diagnosis of NHL. When cases were restricted to latitudes greater than 40° ('north'), seasonal patterns were apparent only for ALL and hepatoblastoma. Notably, 33% of hepatoblastoma cases were diagnosed in the summer months. In contrast, for latitudes less than 40° ('south'), only CNS tumours demonstrated a seasonal pattern ($P$ = 0.002; winter). Although these data provide modest support for a summer peak in the diagnosis of childhood ALL, any underlying biological mechanisms that account for these seasonal patterns are likely complex and in need of more definitive studies. © 1999 Cancer Research Campaign

**Keywords**: seasonality; childhood cancer; leukaemia; infection

Nearly 8000 children are diagnosed with cancer each year in the USA (Bleyer, 1990), and for the vast majority, the aetiology is unknown. Based primarily on the temporal and age-specific patterns for specific childhood malignancies (including acute lymphoblastic leukaemia (ALL), non-Hodgkin's lymphoma (NHL) and Hodgkin's disease (HD)), hypotheses relating to infectious agents have been proposed (Mueller, 1991; Kinlen, 1995; Greaves, 1997; Smith et al, 1998; Westerbeek et al, 1998). For example, childhood ALL demonstrates a peak in incidence between 2 and 5 years of age. This age peak was first observed in the 1930s for US white children and was subsequently noted in the 1960s for US black children. The peak is absent in African countries. Several theories suggest that this age peak may be due to exposure(s) to an infectious agent(s) (reviewed in Ross et al, 1994). Clusters of childhood leukaemia in space and time have also been reported in several countries, and offer some support for an infectious aetiology (Ross et al, 1994). Despite these observations, however, no infectious agent has been definitively linked with childhood ALL. Based largely on these ecological observations suggesting an infectious aetiology, numerous investigators have explored whether seasonal variations exist in the diagnosis month (or birth month) of children with leukaemia (or other related malignancies), with inconsistent results (Hayes, 1961; Lee, 1962, 1963; Fraumeni, 1963; Bjelke, 1964; Knox, 1964; Lanzkowsky, 1964; Mainwaring, 1966; Stark and Mantel, 1967; Till, 1967; Gunz and Spears, 1968; Fekety and Carey, 1969; Walker and Van Noord, 1982; van Steensel-Moll et al, 1983; Harris and Al-Rashid, 1984; Harris et al, 1987; Meltzer et al, 1996; Badrinath et al, 1997; Gilman et al, 1998; Westerbeek et al, 1998).

A demographic database was established in the USA to investigate the geographic distribution of over 20 000 children with cancer diagnosed between 1989 and 1991 in the Children's Cancer Group (CCG) and the Pediatric Oncology Group (POG) (Ross et al, 1993, 1996). This large database provided a unique opportunity to examine whether seasonal variations exist in the diagnosis of childhood ALL, HD, or NHL. Moreover, the large number of cases enabled us to explore potential seasonal variations in the diagnosis of other childhood malignancies.

*Received 15 December 1998*
*Revised 10 February 1999*
*Accepted 22 February 1999*

*Correspondence to:* JA Ross, University of Minnesota Division of Pediatric Epidemiology and Clinical Research, Department of Pediatrics, PO Box 422, 420 Delaware Street SE, Minneapolis, MN 55455, USA

### MATERIALS AND METHODS

Data from this study were derived from information on patients seen at one of the member institutions of the CCG or the POG and have been previously described (Ross et al, 1993, 1996; Swensen et al, 1997). Briefly, data were collected on 21 026 incident

**Table 1** Distribution of childhood cancer cases by month of diagnosis

| Diagnosis | Jan | Feb | Mar | Apr | May | Jun | Jul | Aug | Sep | Oct | Nov | Dec | Roger's test |
|---|---|---|---|---|---|---|---|---|---|---|---|---|---|
| Overall | 1806 | 1674 | 1747 | 1724 | 1824 | 1722 | 1778 | 1916 | 1638 | 1765 | 1763 | 1592 | *P* = 0.12 |
| *n* = 20 949 | (8.6)[a] | (8.0) | (8.3) | (8.2) | (8.7) | (8.2) | (8.5) | (9.2) | (7.8) | (8.4) | (8.4) | (7.6) | |
| ALL | 452 | 398 | 447 | 478 | 492 | 467 | 496 | 495 | 460 | 456 | 467 | 424 | *P* = 0.01 |
| *n* = 5532 | (8.2) | (7.2) | (8.1) | (8.6) | (8.9) | (8.4) | (9.0) | (9.0) | (8.3) | (8.2) | (8.4) | (7.7) | |
| AML | 91 | 94 | 97 | 97 | 86 | 105 | 106 | 113 | 92 | 94 | 87 | 91 | *P* = 0.33 |
| *n* = 1153 | (7.9) | (8.2) | (8.4) | (8.4) | (7.5) | (9.1) | (9.2) | (9.8) | (8.0) | (8.2) | (7.6) | (7.9) | |
| HD | 102 | 84 | 88 | 98 | 98 | 97 | 83 | 101 | 96 | 88 | 111 | 96 | *P* = 0.62 |
| *n* = 1135 | (9.0) | (7.4) | (7.8) | (8.6) | (8.6) | (8.5) | (7.3) | (8.9) | (8.5) | (7.8) | (9.8) | (8.5) | |
| NHL | 107 | 102 | 107 | 107 | 131 | 97 | 131 | 105 | 103 | 116 | 113 | 106 | *P* = 0.71 |
| *n* = 1325 | (8.1) | (7.7) | (8.1) | (8.1) | (9.9) | (7.3) | (9.9) | (7.9) | (7.8) | (8.8) | (8.5) | (8.0) | |
| NB | 141 | 114 | 130 | 129 | 142 | 103 | 129 | 120 | 121 | 114 | 135 | 117 | *P* = 0.42 |
| *n* = 1495 | (9.4) | (7.6) | (8.7) | (8.6) | (9.5) | (6.9) | (8.6) | (8.0) | (8.1) | (7.6) | (9.0) | (7.8) | |
| Rb | 39 | 33 | 32 | 37 | 30 | 34 | 24 | 41 | 29 | 39 | 35 | 29 | *P* = 0.77 |
| *n* = 402 | (9.7) | (8.2) | (8.0) | (9.2) | (7.5) | (8.5) | (6.0) | (10.2) | (7.2) | (9.7) | (8.7) | (7.2) | |
| WT | 90 | 105 | 100 | 107 | 121 | 107 | 113 | 112 | 89 | 119 | 92 | 90 | *P* = 0.08 |
| *n* = 1245 | (7.2) | (8.4) | (8.0) | (8.6) | (9.7) | (8.6) | (9.1) | (9.0) | (7.1) | (9.6) | (7.4) | (7.2) | |
| RD | 62 | 81 | 89 | 68 | 86 | 68 | 65 | 99 | 51 | 53 | 70 | 69 | *P* = 0.03 |
| *n* = 861 | (7.2) | (9.4) | (10.3) | (7.9) | (10.0) | (7.9) | (7.5) | (11.5) | (5.9) | (6.2) | (8.1) | (8.0) | |
| OS | 63 | 70 | 56 | 61 | 67 | 57 | 60 | 84 | 61 | 86 | 61 | 50 | *P* = 0.31 |
| *n* = 776 | (8.1) | (9.0) | (7.2) | (7.9) | (8.6) | (7.3) | (7.7) | (10.8) | (7.9) | (11.1) | (7.9) | (6.4) | |
| ES | 48 | 29 | 37 | 40 | 31 | 39 | 39 | 47 | 32 | 35 | 32 | 28 | *P* = 0.69 |
| *n* = 437 | (11.0) | (6.6) | (8.5) | (9.2) | (7.1) | (8.9) | (8.9) | (10.8) | (7.3) | (8.0) | (7.3) | (6.4) | |
| CNS | 365 | 335 | 325 | 306 | 313 | 309 | 306 | 339 | 286 | 341 | 323 | 307 | *P* = 0.09 |
| *n* = 3855 | (9.5) | (8.7) | (8.4) | (7.9) | (8.1) | (8.0) | (7.9) | (8.8) | (7.4) | (8.9) | (8.4) | (8.0) | |
| HB | 17 | 12 | 21 | 15 | 20 | 32 | 20 | 23 | 22 | 17 | 17 | 12 | *P* = 0.01 |
| *n* = 228 | (7.5) | (5.3) | (9.2) | (6.6) | (8.8) | (14.0) | (8.8) | (10.1) | (9.7) | (7.5) | (7.5) | (5.3) | |

[a]Percentage of total by diagnosis category. ALL=acute lymphoblastic leukaemia; AML=acute myeloid leukaemia; HD=Hodgkin's disease; NHL=non-Hodgkin's lymphoma; NB=neuroblastoma; Rb=retinoblastoma; WT=Wilms' tumour; RD=rhabdomyosarcoma; OS=osteosarcoma; ES=Ewing's sarcoma; CNS =central nervous system; HB=hepatoblastoma.

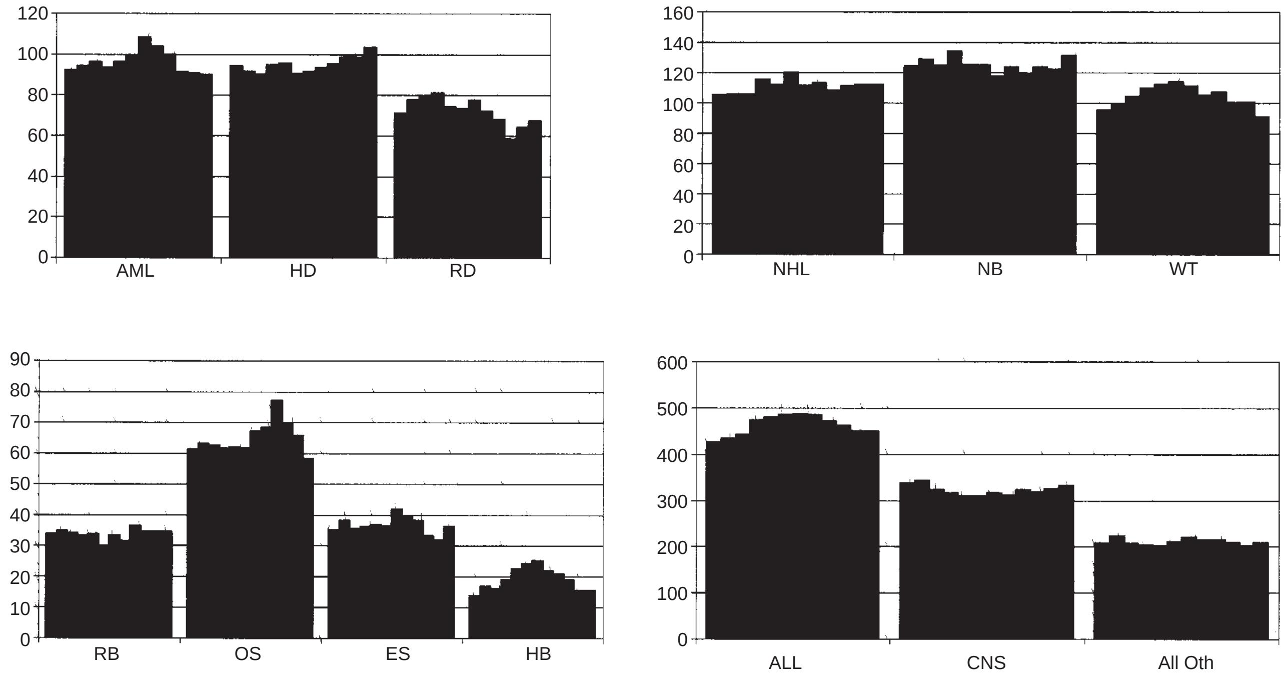

**Figure 1**

 *© 1999 Cancer Research Campaign*

**Table 2** Studies that have explored childhood cancer and seasonality

| Author, year published, place | Data analysed | No. of cases, age group, malignancy | Findings | Statistical test/comment |
|---|---|---|---|---|
| Hayes, 1961, USA | Patients seen at a North Carolina hospital; 1943–1958 | 184 patients with acute leukaemia ages 0–78 years | Excess of cases with clinical onset and/or diagnosis in the winter months | All months ($\chi^2$) |
| Lee, 1962, 1963, England and Wales | National Cancer Registration Scheme | 2130 patients with acute leukaemia ages 0–19 years; 2570 patients with acute leukaemia ages 20–44 years | Summer (May–October) preponderance of ALL for both age groups; slight summer excess of AML in older age group | All months ($\chi^2$) |
| Fraumeni, 1963, USA | National Cooperative Leukemia Study; 1958–1961 | 511 cases < 16 years of age | Winter/spring excess of leukaemia; particularly ALL | All months ($\chi^2$) |
| Bjelke, 1964, Norway | Cancer Registry of Norway, 1953–1958 | 242 children < 20 years of age with leukaemia | No seasonal patterns in month of diagnosis | All months ($\chi^2$) |
| Knox, 1964, England | Northern England; 1951–1960 | < 185 children diagnosed < 15 years of age; acute leukaemia | May–October preponderance of clinical onset in children less than 6 years of age | Summer–winter ratios ($\chi^2$) |
| Lanzkowsky, 1964, South Africa | Paediatric Teaching Unit of the University of Capetown; 1959–1964 | 40 children < 14 years of age | Winter excess (June–August) of childhood acute leukaemia | Visual plots |
| Mainwaring, 1966, London | Diagnosis records from a hospital in Liverpool, 1955–1964 | 74 children < 15 years of age with leukaemia | No seasonal patterns in the date of onset of leukaemia | All months ($\chi^2$) |
| Stark and Mantel, 1967, USA | Michigan death records; 1950–1964 | 706 children < 15 years of age who died of leukaemia | No seasonal pattern in the month of birth of cases | All months ($\chi^2$) |
| Till et al, 1967, UK | London death certificates, 1952–1961 | 618 children < 15 years of age with leukaemia | No seasonal patterns in date of onset | All months ($\chi^2$) |
| Gunz and Spears, 1968, New Zealand | Cancer Death Lists in New Zealand; 1953–1964 | A total of 1003 children and adults with leukaemia | Peak in summer for adults, not for children | All months ($\chi^2$ and goodness of fit test) |
| Fekety and Carey, 1969 | Johns Hopkins Hospital | < 20 years of age; 96 children with acute leukaemia | Late spring/early summer excess in leukaemia onset | Visual plots |
| Walker and Van Noord, 1982, USA | SEER program and TNCS*; 1969–1981 | 7000 cases of leukaemia; all ages; also by leukaemia type | No seasonal patterns for either all leukaemia or for different cell types or age groups | Edward's test; did not explore patterns with respect to geography |
| van Steensel-Moll et al, 1983, Netherlands | Dutch Childhood Leukemia Study Group; 1973–1980 | 293 children < 15 with leukaemia | No seasonal variations by age, sex or leukaemia type | Edwards, Knox and Mantel methods |
| Harris and Al-Rashid, 1984 | 18 hospitals and medical centres in Nebraska, 1971–1980 | 101 children < 15 years of age with ALL | Spring (March, April) and late summer–early fall excess (August, September) of ALL | Analysis of variance |
| Harris et al, 1987 | SEER data + Nebraska; 1973–1980 | 1221 children < 20 with ALL | Statistically significant peaks occurred in April, August and December for cases who resided in US locations above 40°; and in February, July and October for cases who resided in locations below 40° | Periodic regression analysis evaluating four types of sinusoidal curves: unimodal, bimodal, trimodal or quatrimodal; also evaluated above and below 40° latitude |
| Meltzer et al, 1996, USA | SEER data, 1973–1986 | 1487 children < 15 with ALL | No statistically significant association with month of birth and childhood ALL was found | Cosinor analysis; also evaluated geographical regions |
| Badrinath et al, 1997, UK | East Anglian Cancer Registry, 1971–1994 | 338 children < 15; 3954 adults; all leukaemias | Significant 40% summer excess in month of diagnosis for ALL both in children and adults | Summer–winter ratios |
| Gilman et al, 1998, UK | Oxford Survey of Childhood Cancers; 1953–1981 | 9207 children with leukaemia under 15; including 5312 ALL | Little evidence of seasonality for the overall group; suggestion of seasonality (summer peak) in a regional data set (West Midlands) | Summer–winter ratios($\chi^2$) |
| Westerbeek et al, 1998, UK | Manchester Children's Tumour Registry, 1954–1996 | 1070 children < 15 with ALL; 244 children < 15 with AML; 166 children < 15 with HD; 189 children with NHL | Statistically significant winter peak in date of first symptom for common ALL and HD; using date of diagnosis and summer/winter ratios for ALL, a significant summer excess was noted as in the Badrinath study above | Edward's test |

*TNCS= Third National Cancer Survey.

© 1999 Cancer Research Campaign childhood cancer cases (less than 20 years of age) diagnosed in the continental USA (i.e. excluding Hawaii and Alaska) by a member institution of CCG or POG between 1 January 1989 and 31 December 1991. Data collected included date of birth, date of diagnosis, sex, race, diagnosis code and the zip code of residence at the time of diagnosis. Zip codes were used to determine latitude position. For this analysis, a subset of cases ($n$ = 77) had missing or insufficient data (e.g. lacking month) on date of diagnosis or date of birth. Thus, a total of 20 949 cases were evaluated for seasonal variations in the date of diagnosis.

Childhood cancers explored in this study included ALL, acute myeloid leukaemia (AML), HD, NHL, retinoblastoma (Rb), central nervous system (CNS) tumours, rhabdomyosarcoma (RD), hepatoblastoma (HB), Wilms' tumour (WT), osteosarcoma (OS), Ewing's sarcoma (ES) and neuroblastoma (NB).

### Statistical analysis

Seasonal trends (adjusted for the number of days in a month) were evaluated with Roger's test and maximum likelihood estimates (Roger, 1977). Roger's test is a modification of Edward's test and evaluates the significance for cyclic trends based on the efficient score vector calculated for one seasonal peak (Edwards, 1961; Roger, 1977; Epilog, EpiCenter Software). Additional peaks were evaluated by a maximum likelihood model that fits an order 1 model, then order 2, etc. Plots of the number of cases of childhood cancer by month of diagnosis were also examined. Three-month moving means were used in the plots to assist in visual evaluation of seasonal trends.

### RESULTS

Table 1 shows the overall distribution of cases by month of diagnosis, and by diagnosis-specific subgroups. Although there were some months (January, May and August) where there was a notably higher frequency of cases overall, the test for seasonal trend was not significant ($P$ = 0.12). For the individual diagnostic groups, there was a statistically significant summer peak evident for ALL ($P$ = 0.01), RD ($P$ = 0.03) and HB ($P$ = 0.01) (Table 1 and Figure 1). Marginally significant peaks were observed for WT (summer) and CNS tumours (winter) ($P$ = 0.08 and 0.09 respectively). No statistically significant seasonal patterns were detected for AML (although there were notably more cases in the summer), osteosarcoma (notable fall peak), HD, NHL, NB, RB, or all other tumours combined.

When the cases were stratified by northern and southern latitudes, several different patterns were apparent (data not shown). For cases living in the southern USA, with the exception of a winter peak for CNS tumours ($P$ = 0.002), there were no statistically significant seasonal patterns. For cases living in the northern USA, there was a statistically significant summer peak for both ALL ($P$ = 0.004) and HB ($P$ = 0.03).

### DISCUSSION

In an evaluation of over 20 000 children diagnosed with cancer in the USA, we found statistically significant seasonal variations in the diagnosis of several malignancies including ALL (summer peak), HB (summer peak) and RD (spring/summer peak).

A summer excess in the diagnosis of childhood ALL has been reported in several studies (Lee, 1962, 1963; Knox, 1964; Fekety, 1969; Badrinath et al, 1997; Westerbeek et al, 1998) (Table 2). Others have found no seasonal variations in childhood ALL (Bjelke, 1964; Mainwaring, 1966; Till, 1967; Gunz, 1968; Walker, 1982; van Steensel-Moll et al, 1983; Gillman et al, 1998), a winter excess (Hayes, 1961; Fraumeni, 1963; Lanzkowsky, 1964), or a more complex (multiseasonal) pattern (Harris and Al-Rashid, 1984; Harris et al, 1987). Harris et al (1987) compared seasonal risk of childhood ALL in cases diagnosed in the northern USA (greater than 40° latitude) including Seattle, Nebraska, Iowa, Detroit and Connecticut with cases diagnosed in the southern USA (less than 40° latitude) including San Francisco, Utah, New Mexico and Atlanta. They found more complex trimodal patterns, with seasonal peaks in April, August and December for northern cases, and seasonal peaks in February, July and October for the southern locations. They suggest that these peaks could coincide with seasonal elevations in the occurrence of allergic and infectious processes, including a higher frequency of tree and grass pollen in the spring, ragweed in the summer and influenza in the winter. In our study, when cases were stratified on latitude, a summer excess for ALL was only apparent for children who resided in the northern USA; there was no suggestion of a trimodal seasonal pattern. Others who have observed only singular summer peaks in ALL diagnosis also speculate that a cyclic pattern in an infectious exposure may account for the patterns observed (Gunz and Spears, 1968; Badrinath et al, 1997). While it is acknowledged that exact patterns of seasonality in potential infectious agents cannot be easily delineated, attention to the occurrence of infections in children with leukaemia prior to diagnosis in epidemiological studies may be fruitful.

The excess of childhood AML diagnosed in the summer, although not statistically significant, may still be important to note. While ALL and AML are biologically different diseases and may have distinct aetiologies (Ross et al, 1994), there is no reason to think that an infectious component must only be limited to ALL. It is possible that different infectious agents may play a separate role in the development of each of these diseases. This preliminary evidence for a summer peak in AML merits further investigation.

The fall peak in osteosarcoma is visually striking but not statistically significant. We also found summer peaks in the diagnosis of RD, HB and WT. We observed no seasonal patterns among children diagnosed with CNS tumours in the northern states. There did appear to be a winter peak, however, among children in the southern states.

In light of the fact that these observations have not been reported in other populations and that we did not have an a priori hypothesis regarding diagnostic seasonality at these sites, caution should be used in drawing any conclusions based solely on these data.

Several potential limitations of this study need to be considered. First, this was an ecological analysis. We had no information at the individual level on potential confounding factors. Second, a number of different childhood tumours were evaluated for seasonal patterns in this study. While there were a priori hypotheses for some of these (such as ALL and NHL), most of the sites were evaluated on a hypothesis-generating basis. In fact, the finding of a summer peak for multiple sites within this analysis provides some evidence against a causal interpretation of the results for ALL. Third, we did not have information on the month of onset of clinical symptoms. This date may be the more important indicator, especially for ALL, since there may be considerable variability in the time period from first appearance of symptoms to

© 1999 Cancer Research Campaign diagnosis. Fourth, children included in this study were diagnosed over a period of 3 years. It is possible that if the patterns of seasonal diagnosis of a specific cancer are dependent on exposure to infectious agents, then these seasonal patterns might vary from year to year in the same way that infections do. On the other hand, the cyclical patterns involved in exposure to infectious agents might occur over a longer time frame (i.e. greater than 3 years). Fifth, this type of study is unable to evaluate exposure to specific infectious agents. Finally, because the aetiology of most cancers is likely multifactorial, not all cases would necessarily have an infectious cause. This mixing of effects, however, would probably result in an underestimate of any putative seasonal influence.

In summary, this study provides some modest support for a summer excess in the diagnosis of childhood ALL. Given all the available evidence, it seems reasonable to suggest that these data may reflect some underlying pattern of exposure to infectious agents. While no specific agent has yet been identified and associated with the development of childhood ALL, the theories of Greaves (1997), Kinlen (1995) and Smith (Smith et al, 1998) deserve serious attention and investigation. If there are underlying biological mechanisms that account for the seasonal patterns observed here and in other preliminary studies, they are very likely to be complex and difficult to unravel. We suggest that further studies are most likely to be fruitful only if they are based on a more detailed investigation of specific hypotheses relating to specific infectious agents.

### ACKNOWLEDGEMENTS

Supported by a grant from the American Cancer Society, the Children's Cancer Research Fund, NIH grant CA75169 (Dr Ross) and NIH grants CA13539 (Children's Cancer Group), CA29139 and CA37379 (Pediatric Oncology Group).

### REFERENCES

Badrinath P, Day NE and Stockton D (1997) Seasonality in the diagnosis of acute lymphocytic leukaemia. *Br J Cancer* **75**: 1711–1713

Bjelke E (1964) Leukemia in children and young adults in Norway. *Cancer* **17**: 248–255

Bleyer WA (1990) The impact of childhood cancer on the United States and the world. *CA Can J Clin* **40**: 355–367

Edwards JH (1961) The recognition and estimation of cyclic trends. *Ann Hum Genet Lond* **25**: 83–87

Fekety FR and Carey JJH (1969) Season and the onset of acute childhood leukemia. *Maryland State Med J* **11**: 73–77

Fraumeni JF (1963) Seasonal variation in leukaemia incidence (letter to the editor). *Br Med J* **2**: 1408–1409

Gilman EA, Sorahan T, Lancashire RJ, Lawrence GM and Cheng KK (1998) Seasonality in the presentation of acute lymphoid leukaemia (letter to the editor). *Br J Cancer* **77**: 677–678

Greaves MF (1997) Aetiology of acute leukaemia. *Lancet* **349**: 1–5

Gunz FW and Spears GFS (1968) Distribution of acute leukaemia in time and space. Studies in New Zealand. *Br Med J* **4**: 604–608

Hall AJ and Peckham CS (1997) Infections in childhood and pregnancy as a cause of adult disease – methods and examples. *Br Med Bull* **53**: 10–23

Harris RE and Al-Rashid R (1984) Seasonal variation in the incidence of childhood acute lymphocytic leukemia in Nebraska. *Nebraska Med J* **6**: 192–198

Harris RE, Harrell FE Jr, Patil KD and Al-Rashid R (1987) The seasonal risk of pediatric/juvenile acute lymphocytic leukemia in the United States. *J Chron Dis* **40**: 915–923

Hayes DM (1961) The seasonal incidence of acute leukemia. *Cancer* **14**: 1301–1305

Kinlen LJ (1995) Epidemiological evidence for an infective basis in childhood leukaemia. *Br J Cancer* **71**: 1–5

Knox G (1964) Epidemiology of childhood leukaemia in Northumberland and Durham. *Brit J Prev Soc Med* **18**: 17–24

Lanzkowsky P (1964) Variation in leukaemia incidence (letter to the editor). *Br Med J* **1**: 910

Lee JAH (1962) Seasonal variation in the clinical onset of leukaemia in young people. *Br Med J* **1**: 1737–1738

Lee JAH (1963) Seasonal variation in leukaemia incidence (letter to the editor). *Br Med J* **2**: 623

Mainwaring D (1966) Epidemiology of acute leukaemia of childhood in the Liverpool area. *Brit J Prev Soc Med* **20**: 189–194

Meltzer AA, Annegers JF and Spitz MR (1996) Month of birth and incidence of acute lymphoblastic leukemia in children. *Leuk Lymph* **23**: 85–92

Mueller N (1991) An epidemiologist's view of the new molecular biology findings in Hodgkin's disease. *Ann Oncol* **2**: 23–27

Roger JH (1977) A significance test for cyclic trends in incidence data. *Biometrika* **64**: 152–155

Ross JA, Severson RK, Pollock BH, Neglia JP, Woods WG and Hammond GD (1993) Pediatric cancer in the United States. A preliminary report of a collaborative study of the Children's Cancer Group and the Pediatric Oncology Group. *Cancer* **71**: 3415–3421

Ross JA, Davies SM, Potter JD and Robison LL (1994) Epidemiology of childhood leukemia with a focus on infants. *Epidemiol Rev* **16**: 243–272

Ross JA, Severson RK, Pollock BH and Robison LL (1996) Childhood cancer in the United States. A geographical analysis of cases from the Pediatric Cooperative Clinical Trials Groups. *Cancer* **77**: 201–207

Smith MA, Simon R, Strickler HD, McQuillan G, Ries LA and Linet M (1998) Evidence that childhood acute lymphoblastic leukemia is associated with an infectious agent linked to hygiene conditions. *Cancer Causes Control* **9**: 285–298

Stark CR and Mantel N (1967) Temporal-spatial distribution of birth dates for Michigan children with leukemia. *Cancer Res* **27**: 1749–1755

Stewart AM and Kneale GW (1982) The immune system and cancers of foetal origin. *Cancer Immunol Immunother* **14**: 110–116

Swensen AR, Ross JA, Severson RK, Pollock BH and Robison LL (1997) The age peak in childhood acute lymphoblastic leukemia. Exploring the potential relationship with socioeconomic status. *Cancer* **79**: 2045–2051

Till MM, Hardisty RM, Pike MC and Doll R (1967) Childhood leukaemia in greater London: a search for evidence of clustering. *Br Med J* **3**: 755–758

Thorne R, Hunt LP and Mott MG (1998) Seasonality in the diagnosis of childhood acute lymphoblastic leukaemia (letter to the editor). *Br J Cancer* **77**: 678

Van Steensel-Moll HA, Valkenburg HA, Vandenbroucke JP and Van Zanen GE (1983) Time space distribution of childhood leukaemia in the Netherlands. *J Epidemiol Comm Health* **37**: 145–148

Walker AM and Van Noord PAH (1982) No seasonality in the diagnosis of acute leukemia in the United States. *J Natl Cancer Inst* **69**: 1283–1287

Westerbeek RMC, Blair V, Eden OB, Stevens RF, Will AM, Taylor GM and Birch JM (1998) Seasonal variations in the onset of childhood leukaemia and lymphoma. *Br J Cancer* **78**: 119–124

© 1999 Cancer Research Campaign
